# LD-Spline: Mapping SNPs on genotyping platforms to genomic regions using patterns of linkage disequilibrium

**DOI:** 10.1186/1756-0381-2-7

**Published:** 2009-12-03

**Authors:** William S Bush, Guanhua Chen, Eric S Torstenson, Marylyn D Ritchie

**Affiliations:** 1 Center for Human Genetics Research, Department of Molecular Physiology and Biophysics, Vanderbilt University, Nashville, TN, USA; 2 Department of Biostatistics, University of North Carolina, Chapel Hill, NC, USA

## Abstract

**Background:**

Gene-centric analysis tools for genome-wide association study data are being developed both to annotate single locus statistics and to prioritize or group single nucleotide polymorphisms (SNPs) prior to analysis. These approaches require knowledge about the relationships between SNPs on a genotyping platform and genes in the human genome. SNPs in the genome can represent broader genomic regions via linkage disequilibrium (LD), and population-specific patterns of LD can be exploited to generate a data-driven map of SNPs to genes.

**Methods:**

In this study, we implemented LD-Spline, a database routine that defines the genomic boundaries a particular SNP represents using linkage disequilibrium statistics from the International HapMap Project. We compared the LD-Spline haplotype block partitioning approach to that of the four gamete rule and the Gabriel et al. approach using simulated data; in addition, we processed two commonly used genome-wide association study platforms.

**Results:**

We illustrate that LD-Spline performs comparably to the four-gamete rule and the Gabriel et al. approach; however as a SNP-centric approach LD-Spline has the added benefit of systematically identifying a genomic boundary for each SNP, where the global block partitioning approaches may falter due to sampling variation in LD statistics.

**Conclusion:**

LD-Spline is an integrated database routine that quickly and effectively defines the genomic region marked by a SNP using linkage disequilibrium, with a SNP-centric block definition algorithm.

## Background

Recent advances in high-throughput genotyping technology have ushered in the era of genome-wide association (GWA) studies [1]. The GWA approach has seen much success over the last few years, identifying many novel genetic effects for a multitude of human disease phenotypes [2]. The underlying philosophy of this research approach is that a dense panel of single nucleotide polymorphisms (SNPs) can mark broader genomic regions by exploiting patterns of linkage disequilibrium.

Linkage disequilibrium (LD), a term first coined by Lewontin and Kojima in the field of population genetics to describe the non-random association of alleles at multiple loci [3], arises when a mutation occurs near a marker on a common haplotype background [4]. If there is no recombination between the marker and the mutation, the pair is passed together to offspring in subsequent generations. When assayed, the mutation and the marker always appear together in the population, and over time the haplotype carrying the mutation can become common. Eventually, through multiple generations and recombination events, the marker and the mutation are separated by a recombination event in some individuals. As this occurs more and more over successive generations, the LD decays, or approaches *linkage equilibrium*, when the marker and the mutation appear to be independent in the population. The decay of LD, similar in concept to radioactive decay, is directly related to the genetic distance between the two markers (the frequency of recombination events expected between the two).

Numerous phenomena in population genetics and evolutionary biology can impact LD structure [5]. Patterns of mating, geographic subdivision, natural selection, and mutation can all change LD. Genetic drift, for example, can create LD between nearby markers simply by oversampling a multi-marker haplotype. Similarly, population bottlenecks or subdivisions effectively resample an LD structure from the larger population, producing chance haplotype effects, thereby increasing LD [6,7]. Along these lines, various attributes of LD have been exploited to identify regions of positive selection [8].

LD has recently become of great interest to genetic epidemiologists, as patterns of LD first proved useful for fine mapping of disease genes and later for large-scale surveys of much of the human genome. These patterns, which manifest in SNP data as correlations between genotypes of nearby SNPs in the panel, are generally caused as these SNPs on a common genomic background are transmitted through human subpopulations. In such gene mapping studies, associations are classified either as indirect or direct [9]. An indirect association occurs if an influential polymorphism is located on the larger genomic region surveyed by genotyping other SNPs that mark the region. Any genotyped SNPs on the same genomic background as the influential polymorphism would appear associated to the disease being studied. If the influential variant itself is genotyped in the study, it would have a direct association to the phenotype. Generally when a SNP is associated and then sufficiently replicated, the genomic region surrounding this SNP is re-sequenced to identify the true influential variation.

Many measures of LD have been proposed [10], though all are ultimately related to the frequency difference between a two-marker haplotype and the frequency expected assuming that the two markers are independent. The two commonly used measures of linkage disequilibrium are D' and r^2^[10,11] shown in equations 1 and 2. In these equations, π_12 _is the frequency of the ab haplotype, π_1· _is the frequency of the a allele, and π_2· _is the frequency of the b allele.

D' is a population genetics measure that is related to recombination events between markers and is scaled between 0 and 1. A D' value of 0 indicates complete linkage equilibrium, which implies frequent recombination between the two markers and statistical independence under principles of Hardy-Weinberg equilibrium. A D' of 1 indicates complete linkage disequilibrium, indicating no recombination between the two markers. Alternatively, r^2 ^is the square of the correlation coefficient, and is a more statistical measure of shared information between two markers. The r^2 ^measure is commonly used to determine how well one SNP can act as a surrogate for another. There are multiple dependencies between these two statistics [5,10], but most notably r^2 ^is sensitive to the allele frequencies of the two markers, and can only be high in regions of high D'.

LD measures are based (at some level) on a two-marker haplotype frequency. One often forgotten issue associated with LD measures is that current technology does not allow direct measurement of these frequencies from a sample because each SNP is genotyped independently, and the *phase*, or chromosome of origin for each allele, is unknown. Many well developed and documented methods for inferring haplotype phase and estimating the subsequent two-marker haplotype frequencies exist [12], and generally lead to reasonable results [13].

The International HapMap Project cataloged distinct patterns of LD in four human sub-populations: Yoruba, Caucasian, Han Chinese, and Japanese [11]. Phase I of this project examined 2.5 million SNPs across the human genome, computing pair-wise D' and r^2 ^statistics in 500 KB windows. These values were made publicly available as flat-file downloads from the HapMap project, release 21. Phase III of the HapMap project expands the available populations to include Toscans from Italy, Luhya and Maasai from Kenya, and US individuals with African and Mexican ancestry.

Enrichment analysis of GWA single marker results is a common procedure to examine the functional relationships between genes in the significant marker set. This approach, and many new bioinformatics and statistical techniques, take a gene-centric approach to analysis. Aubert et al. proposed a gene-based local false discovery rate (FDR) procedure [14]. Li et al. proposed prioritizing SNPs within candidate genes in genome-wide scans to improve power, using an FDR analysis on result subsets [15]. Lewinger et al. and Province and Borecki proposed elegant pathway-based Bayesian approaches to GWA analysis, incorporating gene information into SNP analysis [16,17]. Because these techniques require relating SNPs on a genotyping platform to genes in the genome, a systematic and user-controlled method for mapping SNPs to the broader genomic regions they mark - and ultimately to genes - is needed.

While the simplest approach for generating SNP-gene relationships is to determine if a SNP lies within the exonic or intronic region outlined by an annotated genomic build, some approaches pad the gene boundaries with a user-defined region (50 KB for example) upstream and downstream to account for possible linkage disequilibrium and/or regulatory regions (see methods of [18]). There are also several approaches for generating LD statistics that can then be used to partition genomic regions captured by genotyping platforms. The popular PLINK software has two options for generating LD information [19,20]. r^2 ^descriptive statistics can be determined quickly and simply by computing correlations between genotypes. Inferential statistics such as population estimates of D' and r^2 ^can also be computed by PLINK, but this procedure is much more computationally costly as it requires phasing haplotypes. LdCompare is another approach that can rapidly compute pair-wise r^2 ^values from genotype data, generating multi-marker correlations when given phased data [21]. While these approaches provide valuable information about the redundancy of information captured by a genotyping platform, they do not readily relate a single SNP from the platform to the larger genomic region it potentially represents - that must be accomplished by a post-processing step.

Currently, haplotype blocks are generally identified using two approaches, the Gabriel et al. method and the four gamete rule. These two approaches are implemented in Haploview software, and produce a global haplotype block partition for a given set of SNP genotypes. Both procedures are sequential, beginning with the first SNP in the dataset and defining non-overlapping blocks upstream. While these approaches provide the general haplotype structure of a given genomic region, they are *global *rather than *SNP-centric *procedures. These approaches could misrepresent the genomic region a particular SNP marks based on the global sequential nature of the partitioning strategy.

In this work, we present an algorithm that systematically relates SNPs to genes or genomic regions by processing pair-wise LD statistics. This algorithm is implemented as a MySQL aggregate function and performs genomic region and gene assignments for collections of SNPs, such as GWAS SNP marker lists, using locally stored LD information from the International HapMap Project.

## Results

### Algorithm

**Figure 1:**
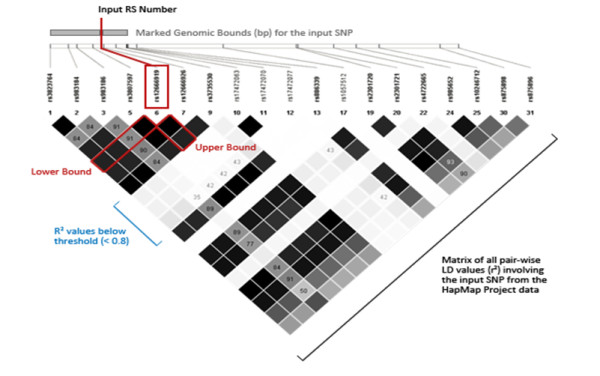
**Overview of the LD-Spline Algorithm**. A matrix of all HapMap-based pair-wise LD values (D' or r^2^) is retrieved from the database. Using this matrix, the lower bound is incrementally extended to downstream SNPs while the pair-wise LD value between the downstream SNP and the input SNP is greater than the user-defined threshold (in this case r^2 ^> 0.8). The process is repeated for the upper bound to define the marked genomic bounds for the input SNP.

To execute the LD-Spline function, a user specifies the following: the LD statistic to be used (D' or r^2^), an LD statistic threshold value (ranging between 0 and 1), and a reference sequence (RS) SNP identifier. The RS ID is used to query the specified LD statistic for all pair-wise values that exist in the HapMap data that include the specified SNP. The procedure is illustrated in figure 1 and outlined in algorithm 1, and can be applied to LD values corresponding to any population.

#### Algorithm 1

Input: RS number of the SNP to map (rs_id), table or matrix of pair-wise LD values

1. Initialize the upper and lower bounds of the marked genomic region with the position of the input SNP.

2. Retrieve the value of the selected LD measure corresponding to the input SNP and the next downstream SNP, SNP X.

3. If the LD value is greater than the threshold value, change the lower bound of the marked genomic region to the position of SNP X.

4. Repeat 3 and 4 to extend the lower bound until the retrieved LD value is less than the threshold value.

5. Repeat 2 - 4 to define the upper bound.

### Testing

**Figure 2:**
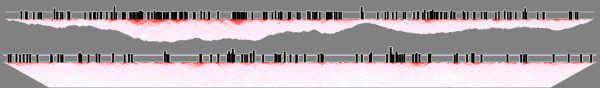
**Linkage disequilibrium (D') of chromosome 1 (top) and chromosome 18 (bottom) simulated using genomeSIMLA**. Haploview-style correlation plots illustrate the LD structure (in D'). Each black line above the correlation plot indicates a haplotype block generated by the simulation, and the height of the bar above the horizontal line indicates SNP density.

**Figure 3:**
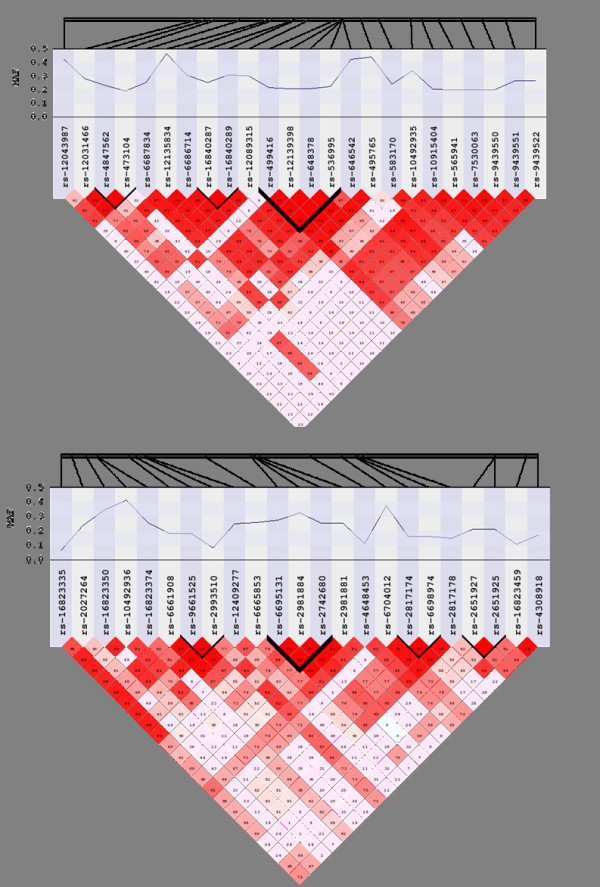
**Regional haplotype structure for simulated block 7 (top) and 5 (bottom) on chromosome 1**. The physical location and minor allele frequency of each simulated SNP is shown on the tracks along the top of the figure, and LD structure in D' is shown in a Haploview-style correlation plot at the bottom. True haplotype blocks in the population are marked with dark lines in the correlation plot.

An overview of the linkage disequilibrium present in our simulated population is shown in figure 2. The parameters used in this simulation recapitulate reasonable patterns of linkage disequilibrium, similar to those seen in Hapmap data [22]. A more detailed view of two simulated haplotype blocks on chromosome 1 is shown in figure 3. The blocks selected for evaluation ranged in SNP density from 5 SNPs to 2 SNPs.

**Figure 4:**
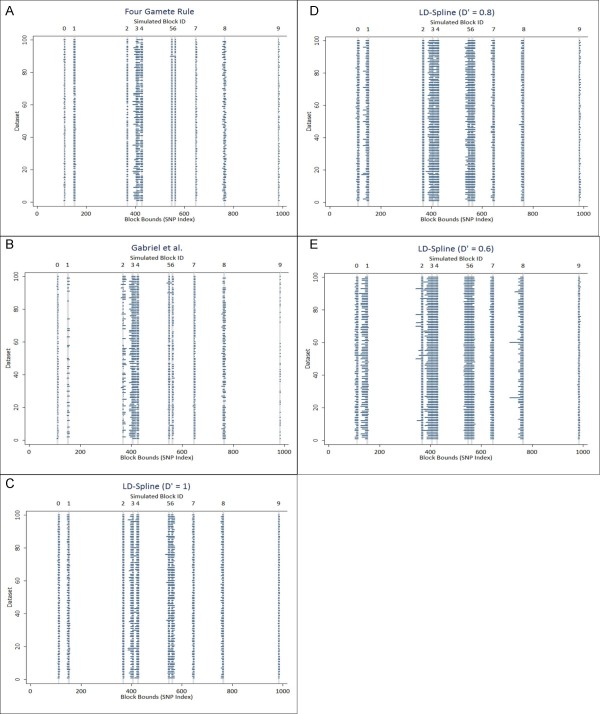
**a-e: Haplotype block partitioning for simulated chromosome 1**. Ten haplotype blocks were selected from the simulation of chromosome 1 for algorithm assessment. Blocks are identified by an integer ID shown across the top of the figure, indicating relative position within the 1000 SNPs simulated. The true bounds for each block are shown as gray vertical lines, with the thickness of the line indicating the block size. Each horizontal line represents a haplotype block called by the four gamete rule(a), Gabriel et al. method(b), or LD-Spline using a D' threshold of 1(c), 0.8(d), or 0.6(e) with the length of the line representing the number of SNPs included in the haplotype block call. The x-axis illustrates the upper and lower SNP index in the dataset for each block, and the y-axis indicates the dataset for which each block is called.

**Figure 5:**
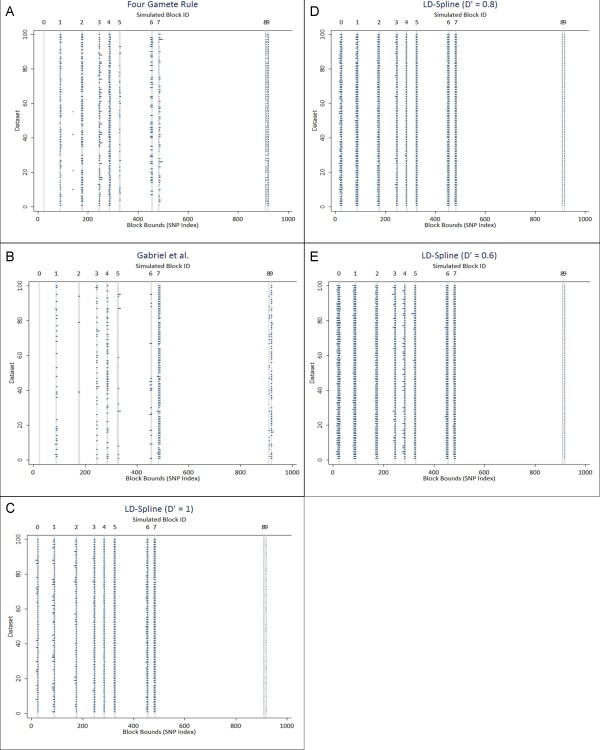
**a- e: Haplotype block partitioning for simulated chromosome 18**. Ten haplotype blocks were selected from the simulation of chromosome 18 for algorithm assessment. Blocks are identified by an integer ID shown across the top of the figure, indicating relative position within the 1000 SNPs simulated. The true bounds for each block are shown as gray vertical lines, with the thickness of the line indicating the block size. Each horizontal line represents a haplotype block called by the four gamete rule(a), Gabriel et al. method(b), or LD-Spline using a D' threshold of 1(c), 0.8(d), or 0.6(e), with the length of the line representing the number of SNPs included in the haplotype block call. The x-axis illustrates the upper and lower SNP index in the dataset for each block, and the y-axis indicates the dataset for which each block is called.

Haplotype block partitioning of 100 datasets from the simulated region of chromosome 1 using each of the evaluated algorithms is shown in figure 4 and chromosome 18 in figure 5. Each horizontal line on these figures represents a called haplotype block, with the x-axis representing the index position of the SNP and the y-axis denoting the dataset for which the block partition was called. The ten gray vertical lines represent the true haplotype blocks simulated in the data (indexed across the top of the figure).

For chromosome 1 (figure 4), note the differences for blocks 3 and 4. The four gamete rule (4a) and the Gabriel et al. method (4b) call these two blocks as one larger block, and the four gamete rule seems more prone to produce a truncated block that does not include both the simulated blocks. LD-Spine (4c-4e) does a better job of separating these two blocks, but is more likely to combine blocks 5 and 6 than Gabriel et al. and the four gamete rule. For chromosome 18 (figure 5), the general block calling from the four gamete rule (5a) and Gabriel et al. (5b) is sparse across datasets, indicating that for this particular simulated chromosome, sampling variability between datasets reduces the ability to find blocks consistently.

Weighted Kappa statistics for inter-rater agreement were calculated pair-wise to compare all algorithms to each other and to the true simulated block bounds. Results for chromosome 1 and chromosome 18 are shown in table 1. All algorithms had statistically significant agreement with each other and with true bounds by z-test [23]. The four-gamete rule performed best, with a weighted kappa near 0.95 for both simulations. The Gabriel et al. approach performed nearly as well. Of the three D' thresholds evaluated in this simulation, using a threshold of 1 best matched the two established algorithms and the true block bounds in the simulation. While the LD-Spline approach does not outperform either of the established algorithms, it performs nearly as well, and still shows excellent agreement with true block bounds.

**Table 1:** Weighted kappa statistics for algorithm agreement.

**Chromosome 1**					
	
	**Four Gamete Rule**	**Gabriel et al**.	**LD-Spline 0.6**	**LD-Spline 0.8**	**LD-Spline 1**
	
**True Bounds**	0.9512	0.9514	0.9092	0.9089	0.9383
**Four Gamete Rule**		0.9762	0.9123	0.9163	0.9498
**Gabriel et al**.			0.8931	0.9054	0.9412
**LD-Spline 0.6**				0.9681	0.9335
**LD-Spline 0.8**					0.9479
					
**Chromosome 18**					
	
	**Four Gamete Rule**	**Gabriel et al**.	**LD-Spline 0.6**	**LD-Spline 0.8**	**LD-Spline 1**
	
**True Bounds**	0.9566	0.9271	0.9377	0.9153	0.9374
**Four Gamete Rule**		0.9740	0.9400	0.9379	0.9495
**Gabriel et al**.			0.9226	0.9208	0.9292
**LD-Spline 0.6**				0.9864	0.9635
**LD-Spline 0.8**					0.9671

**Figure 6:**
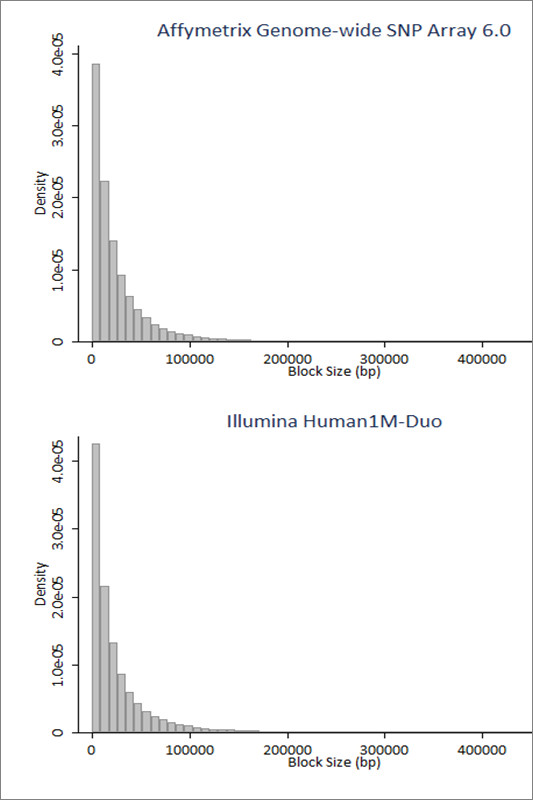
**Frequency histogram of LD-Spline called haplotype block sizes**. The Affymetrix Genome-wide SNP Array 6.0 (top) and the Illumina Human 1M -Duo (bottom) genotyping platforms were processed using the LD-Spline algorithm. The density distribution of haplotype block sizes is shown by frequency histograms.

The LD-Spline algorithm using a D' threshold of 1 was found to best recapitulate true haplotype block boundaries and best match established algorithm block calls. We used these parameters to execute the LD-Spline procedure on two common GWA genotyping platforms, the Affymetrix Genome-Wide SNP Array 6.0 and the Illumina Human1M-Duo BeadChip. Block boundaries were mapped to NCBI genome build 36 using the Ensembl database [24]. Density histograms of haplotype block sizes marked by each genotyping platform are shown in figure 6.

The average block size captured by the Affymetrix 6.0 is 43 KB, and the average block size captured by the Illumina Human1M-Duo is 38 KB. To quantify the number of genes captured by each platform, we used the Ensembl database to identify gene regions (defined as the start of the 5' UTR to the end of the 3' UTR), and to determine whether SNPs lie within this region.). We then identified SNPs from genotyping platforms that fall directly within these gene boundaries. Using this process, 17,418 genes were captured by the Affymetrix 6.0 platform, and 21,024 genes were captured by the Illumina Human 1M platform. Using the marked genomic regions generated by LD-Spline (using a D' threshold of 1), we declare a gene "captured" if the marked region starts, ends or lies completely within the genic region, or alternatively, if the marked region completely encompasses the gene region. Using LD-Spline, the Affymetrix 6.0 captures 29,421 genes and the Illumina Human 1M captures 29,611 genes. Thus, using LD-Spline leads to a 69% and 41% increase in the number of genes covered by Affymetrix and Illumina, respectively.

### Implementation

The LD-Spline algorithm was implemented in C++ as an aggregate function for the MySQL database management system. The aggregate function, ldspline is executed twice; once to define the upper bound and once to define the lower bound. These results are joined to produce the full mapped genomic region for a SNP or set of SNPs. The ldspline function accepts four arguments: a SNP index, an LD measure (either dprime (D') or rsquared (r^2^), a threshold value (between 0 and 1), and a flag value to indicate an upper bound (0) or lower bound (1) search. Let us define a table 'CEU' that contains pair-wise D' and r^2 ^statistics downloaded, inserted, and indexed by a composite key - a pair of indices that reference the two SNPs for which the LD values apply. Let us also define a table 'index_2_rs' that relates a SNP index to an RS number, and a single value, 'PlatformSNP' as the RS number of a SNP on a genotyping platform that we wish to relate to a genomic region. The SQL statement to map this SNP using an r^2 ^threshold of 0.8 would be:

SELECT lower_bound, position, upper_bound FROM

(SELECT ldspline(A.pos2, A.rsquared, 0.8, 0, A.pos1) as upper_bound, A.pos1 AS position FROM

(SELECT * FROM CEU inner join

(select pos from index_2_rs on rs_id = PlatformSNP) as f

where CEU.pos1 = f.pos

) AS A GROUP BY position) AS C

NATURAL JOIN

(SELECT ldspline(B.pos1, B.rsquared, 0.8, 1, B.pos2) as lower_bound, B.pos2 AS position FROM

(SELECT * FROM LD.CEU inner join

(select pos from index_2_rs on rs_id = PlatformSNP) as g

where LD.CEU.pos2 = g.pos

) AS B GROUP BY position) AS D;

Instead of mapping a single SNP, we could instead choose to map the entire platform of SNPs with one statement. In this case, let us define a table 'Genotyping_Platform' that contains an indexed set of RS IDs. The SQL statement to map the entire table of SNPs using a D' threshold of 0.9 would be:

SELECT lower_bound, position, upper_bound FROM

(SELECT ldspline(A.pos2, A.dprime, 0.9, 0, A.pos1) as upper_bound, A.pos1 AS position FROM

(SELECT * FROM CEU inner join

(select pos from Genotyping_Platform a inner join LD.index_2_rs b on a.rs_id = b.rs_id) as f

where CEU.pos1 = f.pos

) AS A GROUP BY position) AS C

NATURAL JOIN

(SELECT ldspline(B.pos1, B.dprime, 0.9, 1, B.pos2) as lower_bound, B.pos2 AS position FROM

(SELECT * FROM LD.CEU inner join

(select pos from Genotyping_Platform a inner join LD.index_2_rs b on a.rs_id = b.rs_id) as g

where LD.CEU.pos2 = g.pos

) AS B GROUP BY position) AS D;

Processing a table of approximately 600,000 SNPs using the user-defined aggregate function has a runtime of approximately 36 hours on a dual Xeon 3.06 GHz machine with 2 GB of RAM.

For ease of evaluation, we also produced a command-line version of this algorithm using the Perl scripting language. This version is functionally equivalent to the MySQL aggregate function, but rather than accessing a database table of pair-wise LD values, it reads LD values from a flat file.

## Discussion

In this work, we introduce LD-Spline, an efficient database procedure for establishing genomic regions that a SNP potentially represents by mining linkage disequilibrium statistics available from the International Hapmap Project. The two established block-calling algorithms [25] and four gamete rule) function by producing a global haplotype block partitioning, starting at the first SNP and sequentially defining blocks upstream. The LD-Spline approach is SNP-centric, in that it uses LD statistics between a user-provided SNP (such as one from a genotyping platform) and surrounding SNPs in the genome to define the region the specified SNP marks. This SNP-centric approach also has a computational advantage, since only relevant haplotype blocks (the region surrounding SNPs of interest) are called by the algorithm. Gabriel et al. and the four gamete rule would require processing and partitioning the entire human genome to determine the regions marked by a genotyping platform. LD-Spline also has the great advantage of running as a fast and efficient self-contained procedure within the database management system, allowing seamless integration with existing database queries and operations.

We compared the LD-Spline algorithm to the Gabriel et al. and four gamete rule methods, and then compared all methods to the true simulated haplotype block boundaries. Weighted kappa agreement statistics between LD-Spline, traditional block calling algorithms, and the true block partition in simulated data were rather good (> 0.90 in most cases). While none of the block partitioning algorithms perfectly identifies true block boundaries, the LD-Spline approach using a D' threshold of 1 appears to work as well as other established algorithms. Reducing this D' threshold below 1.0 effectively maps a SNP to a larger genomic region, and may be useful for identifying more extreme bounds of possible haplotype blocks. This would provide a more liberal interpretation of the region a particular SNP may represent.

As a SNP-centric approach, LD-Spline has the added advantage of consistently marking a genomic region for each SNP. The sequential partitioning achieved by the Gabriel et al. and four gamete rule approaches do not consistently identify a haplotype block for each dataset. For example with chromosome 18, the four gamete rule and Gabriel et al. did not define haplotypes for simulated blocks 0, 2, 5, or 6. For the specific application of determining what genomic region a typed SNP likely represents, a SNP-centric approach is advantageous because the long-range LD patterns specifically related to the typed SNP are exploited. Since sequential partitioning approaches generally use a two-SNP sliding window to define haplotype blocks, they are not robust to situations where short-range LD is weaker than long-range LD. It is important to note that the weighted Kappa statistics for algorithm agreement do not take into account the number of uncalled haplotype blocks, but do indicate that the boundaries for the called haplotype blocks are similar. With this in mind, LD-Spline provides superior performance when assigning genomic regions to typed SNPs because of its SNP-centric nature.

Sampling variability may also explain the lack of block identification for chromosome 18 and the general small degree of disagreement with the true block boundaries. In our data simulations, we empirically track recombination events to produce exact LD statistics and LD block boundaries on the population level. Each simulated dataset was drawn from that population, so sampling variability could lead to biased LD estimates and subsequent block partitions. Also, to more closely mimic real data collection in the HapMap project, datasets were produced as unphased genotype data. We then used Haploview software to estimate two-marker haplotypes using the EM algorithm to calculate D' and r^2 ^LD statistics. This process could also introduce bias and error into the haplotype block calling procedures.

When applied to GWAS genotyping platforms, block sizes follow the pattern expected based on previous estimates of block size by the HapMap project [11]. The average block size does differ slightly between platforms, which could be because of bias in SNP selection by the genotyping platform manufacturers, particularly Illumina [26]. If SNPs are specifically selected that tag larger genomic regions while SNPs in regions of sparse LD are avoided, the average block size could become inflated. Also, if SNPs in genic regions are overrepresented by genotyping platforms, the higher r^2 ^measures that have been found in genic versus inter-genic regions [27] could also lead to inflation. The dramatic increase in the number of genes "captured" by the Affymetrix 6.0 and Illumina Human 1M platforms illustrate that these modern products were designed with linkage disequilibrium patterns in mind, and gene-centric analysis approaches that do not account for LD are likely not using data available for large numbers of genes.

## Conclusion

Overall, we have illustrated the performance of the LD-Spline routine, and the utility of applying this database-centric procedure to GWAS platforms. One key advantage of the database-centric nature of the LD-Spline user-defined function is its easy incorporation into more sophisticated queries for information retrieval. Once established, the database routine can seamlessly extend the range of data queries to include statistics based on a broader genomic region, rather than a single base-pair location.

## Methods

### Data Simulation

**Figure 7:**
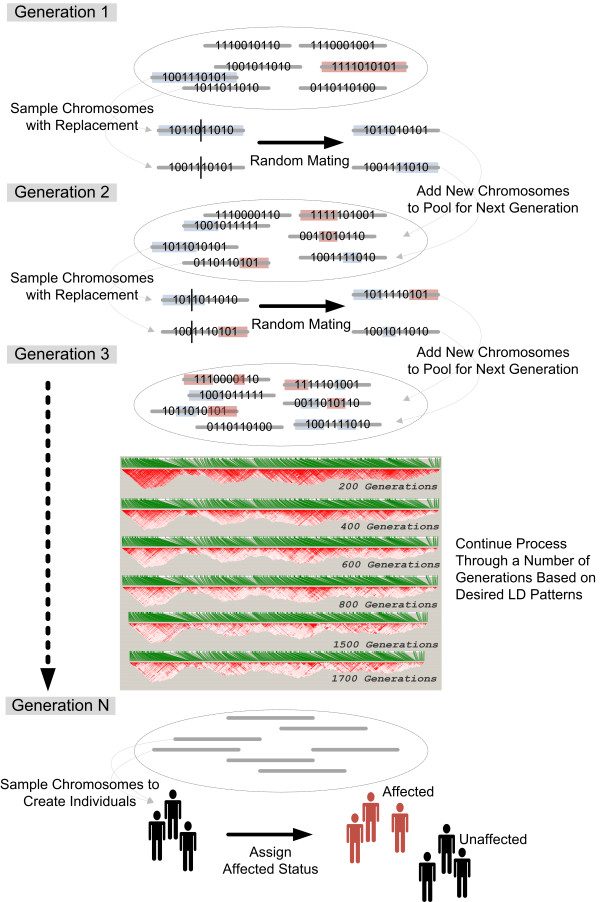
**Overview of the genomeSIMLA process**. Chromosomes are randomly initialized in the first generation, and then randomly sampled with replacement and crossed to produce the next generation. This process continues until the population has the desired LD patterns. Individuals are then sampled from this population for datasets.

We simulated realistic patterns of linkage disequilibrium to mimic two human CEU chromosomal regions using genomeSIMLA (genomeSIM version 2.0.4 software, functionally equivalent to genomeSIMLA 1.0 for LD generation) [22]. GenomeSIMLA is a forward-time population simulator that uses random mating, genetic drift, recombination, and population growth to produce SNP genotype data with linkage disequilibrium. The general procedure for generational advancement is shown in figure 7.

Simulated chromosomes were initialized using random allele frequencies. 1367 SNPs from chromosome 1 were selected from 792,429 bp to 9,965,572 bp, and 1146 SNPs from chromosome 18 were selected from 23,719,514 bp to 24,217,521 bp. All simulated SNPs were included in the HapMap CEU dataset, and HapMap build 35 positional information for each SNP was used. In the simulation, recombinant gametes are created by sampling chromosomes with replacement from the population and crossing over based on intermarker recombination probabilities are determined by the Kosambi function map distance based on a one centimorgan per one million bases of physical distance. The number of recombination events per gamete is drawn from a Poisson distribution. Two gametes are combined to form a new individual for the next generation. This mating and recombination process continues for a user-specified number of generations, the size of each generation is determined by a logistic growth model.

The initial population size was 750, and was advanced over 454 generations using the Richard's growth curve (A = 750, B = 0.02, C = 1,200,000, M = 500, T = 0.01, Var = 0.03) to produce a final population size of 100,000 chromosomes. These parameters are a slight variation on an optimal set described in [22]. Once this population was generated, we produced 100 datasets consisting of 2,000 controls (a null genetic model was used). The random seed for these simulations was 2,225. For this simulated population, we manually selected 10 haplotype blocks and recorded their upper and lower bounding SNPs. GenomeSIMLA tracks recombination events through generational advancement of a population, so the exact haplotype blocks are reported by the simulation. GenomeSIMLA also reports exact D' and r^2 ^statistics computed for the entire population.

### Block Definition Algorithms

In addition to the LD-Spline approach, we evaluated two block calling algorithms implemented in the popular Haploview software [28]: the Gabriel et. al approach [25] and the four-gamete rule [28]. Gabriel et al. used the 95% confidence intervals of D' estimates to establish stretches of "strong LD" [25]. D' estimates are unstable when sample size is small or allele frequency is low, so the confidence intervals of the statistic are used. If the D' 95% confidence upper bound is > 0.98 and the lower bound is > 0.7, there is little statistical evidence of a historical recombination event between the two markers, meaning they form a haplotype block. Alternatively, the four-gamete rule is based on an algorithm described by Wang et al. where the frequency of the four possible two-marker haplotypes are computed for each pair of SNPs [29]. Rather than estimating D' confidence intervals, the four-gamete rule is similar to estimating a confidence interval on the two-marker haplotype frequencies. If all four haplotypes are observed with at least a frequency of 0.01, a recombination event between the two markers likely occurred. These two algorithms were applied to simulated unphased datasets, and the resulting haplotype block partitioning was recorded.

Block partitions were defined by these two algorithms, and compared to three parameterizations of the LD-Spline algorithm: D' threshold of 0.6, D' threshold of 0.8, and D' threshold of 1. For each data simulation, a SNP that lies within each of the 10 selected haplotype blocks was randomly chosen. The LD-Spline approach used these SNPs as input for the algorithm, and haplotype blocks were defined around these SNPs. The Haploview-based algorithms were used to produce a full list of haplotype blocks for each dataset. Once this list was parsed to identify haplotype blocks that contain the randomly selected SNPs, the bounds for those blocks were recorded.

### Algorithm Comparisons

The upper and lower bound SNP indices were compared to the true block boundaries for each block partitioning algorithm using weighted Kappa statistics to assess inter-rater (algorithm) agreement [30]. Weights for the Kappa statistic were calculated using a standard weighting strategy shown in equation 3, incurring an increased penalty as the number of SNPs from the correct boundary edges increased.

In equation 3, *i *is a row index and *j *is a column index of the boundaries specified by the two algorithms, and *k *is the maximum number of possible boundaries the algorithm could call.

The full weighted Kappa statistic is shown in equation 4 [23]. Agreement was evaluated within each of the 10 simulated haplotype blocks and for the overall block partitioning over 100 datasets. Kappa statistics were calculated using STATA 10.

## Software Availability

The LD-Spline software is open-source and freely available from the following website: http://chgr.mc.vanderbilt.edu/ritchielab/LD-Spline

## Competing interests

The authors declare that they have no competing interests.

## Authors' contributions

WB conceived of the study, designed the algorithm, conducted data analysis, and drafted the manuscript. ET implemented and tested the LD-Spline user-defined function for the MySQL database system. GC conducted the data simulation and block partitioning experiments. MR participated in study design, algorithm development, and data analysis, and helped to draft the manuscript. All authors read and approved the final manuscript.
